# An RCT assessing the effects of varying exposure times of therapeutic communication robots on dementia in group homes in Japan

**DOI:** 10.1002/alz.087203

**Published:** 2025-01-09

**Authors:** Kaoru Inoue, Mitsunobu Kono, Ryuji Kobayashi, Takanori Shibata, Chiyomi Yatsu, Daryl Patrick Gamboa Yao, Masahiro Shigeta

**Affiliations:** ^1^ Tokyo Metropolitan University, Arakawa ward, Tokyo Japan; ^2^ Kinjo University, Hakusan city, Ishikawa pref. Japan; ^3^ Hyogo Medical University, Kobe city, Hyogo pref. Japan; ^4^ National Institute of Advanced Industrial Science and Technology, Tsukuba city, Ibaraki pref. Japan; ^5^ University of Illinois at Chicago, Chicago, IL USA; ^6^ The Jikei University School of Medicine, Minato ward, Tokyo Japan

## Abstract

**Background:**

As Japan experiences a super‐ageing society and caregiver manpower decreases, interest in the use of communication robots for active dementia care rises. In the U.S., the communication robot (PARO) has been used as a medical device for psychophysiological biofeedback therapy. We report the effects of varying the exposure time for (PARO) on BPSD severity in people with dementia and on the caregivers' burden of professional caregivers in group homes.

**Methods:**

Cluster randomized controlled trial design. Group homes were randomly assigned to two groups, one using (PARO) once a week and the other thrice a week for one hour per session. For the intervention, (PARO) was placed on a table in a communal area, and participants interacted freely with (PARO). The caregivers provided no encouragement. Changes in BPSD severity and caregivers' burden before and after the intervention were compared using a linear mixed model. The primary outcomes were the severity score and caregivers' burden score of the Neuropsychiatric Inventory brief Questionnaire (NPI‐Q).

**Results:**

Ninety‐one participants were recruited, 85 were included in the analysis. The thrice‐weekly group showed improvement in severity scores before and after the intervention, but there was no statistically significant difference between the once‐weekly and thrice‐weekly groups. The caregivers' burden score was significantly improved in the thrice‐weekly group compared to the once‐weekly group.

**Conclusions:**

Using (PARO) once a week for 1 month did not have a significant effect on either severity or caregivers' burden, whereas thrice a week contributed to reducing caregivers' burden. The effect was better scored on the severity in the thrice‐weekly group than that in the once‐a‐week group, but the difference was not statistically significant, suggesting the need to revisit this issue with a longer intervention period. The impact on caregivers may be evident after a relatively short period. The introduction of (PARO) potentially reduces caregivers’ burden and helps to improve the quality of care, hence providing operational support to group homes.

